# Detection of non-tuberculosus mycobacteria (NTMs) in lung samples using 16S rRNA

**DOI:** 10.1590/0074-02760220031

**Published:** 2022-07-29

**Authors:** Franciele Costa Leite Morais, Graziele Lima Bello, Cíntia Costi, Karen Barros Schmid, Tainá dos Santos Soares, Regina Bones Barcellos, Gisela Unis, Claudia Fontoura Dias, Pedro Eduardo Almeida da Silva, Maria Lucia Rossetti

**Affiliations:** 1 Universidade Luterana do Brasil, Programa de Pós-Graduação em Biologia Celular e Molecular Aplicada à Saúde, Canoas, RS, Brasil; 2 Instituto Nacional de Ciência e Tecnologia em Tuberculose, Programa Institutos Nacionais de Ciência e Tecnologia, Porto Alegre, RS, Brasil; 3 Secretaria da Saúde do Rio Grande do Sul, Centro de Desenvolvimento Científico e Tecnológico, Porto Alegre, RS, Brasil; 4 Universidade Federal do Rio Grande do Sul, Programa de Pós-Graduação em Biologia Celular e Molecular, Porto Alegre, RS, Brasil; 5 Universidade Federal do Rio de Janeiro, Programa de Pós-Graduação em Clínica Médica, Rio de Janeiro, RJ, Brasil; 6 Secretaria da Saúde do Rio Grande do Sul, Hospital Sanatório Partenon, Porto Alegre, RS, Brasil; 7 Universidade Federal do Rio Grande, Faculdade de Medicina, Centro de Pesquisas em Microbiologia Médica, Rio Grande, RS, Brasil

**Keywords:** non-tuberculous mycobacteria (NTMs), DNA, real-time PCR (qPCR)

## Abstract

**BACKGROUND:**

Non-tuberculous mycobacteria (NTMs) cause diseases known as mycobacteriosis and are an important cause of morbidity and mortality. The diagnosis of pulmonary disease caused by NTM is hampered by its clinical similarity with tuberculosis (TB) and by the lack of an accurate and rapid laboratory diagnosis.

**OBJECTIVES:**

Detect DNA from NTMs directly from lung samples using real-time polymerase chain reaction (qPCR) for amplification of 16S rRNA. Additionally, DNA sequencing (*hsp65* and *rpoB* genes) was used to identify the species of MNTs.

**METHODS:**

A total of 68 sputum samples (54 with suspected NTMs and 14 with TB) from patients treated at a referral hospital were used.

**FINDINGS:**

Of these, 27/54 (50%) were qPCR positive for NTMs and 14/14 TB patients (controls) were qPCR negative with an almost perfect concordance (*Kappa* of 0.93) with the *Mycobacterium* spp. culture. Sequencing confirmed the presence of NTM in all positive samples. The most common species was *Mycobacterium gordonae* (33%), followed by *Mycobacterium abscessus* (26%), *Mycobacterium fortuitum* (22%), *Mycobacterium avium* (15%) and *Mycobacterium peregrinum* (4%).

**MAIN CONCLUSIONS:**

The qPCR technique for detecting NTMs targeting 16S rRNA has the potential to detect NTMs and rapidly differentiate from *Mycobacterium tuberculosis*. However, it is necessary to identify the species to help in the differential diagnosis between disease and contamination, and to guide the choice of the therapeutic scheme.

Non-tuberculous mycobacteria (NTMs) consist of species of the genus *Mycobacterium* spp., which do not belong to the *Mycobacterium tuberculosis* complex (that causes tuberculosis - TB). They are present in nature and can eventually cause diseases, including pulmonary ones, with symptoms similar to those of TB, especially in immunosuppressed people, such as those with HIV.^1^ ^,^ ^2^ Despite the high morbidity and mortality, the lack of compulsory notification of mycobacteriosis makes NTMs prevalence data scarce.^3^ Therefore, an increase in prevalence has been reported in different regions of the world, including Brazil.^4^ ^,^ ^5^

Lung infections are the most common disease triggered by NTMs, as they are often associated with structural changes in the lung, such as chronic obstructive pulmonary disease, sequelae of previous pulmonary TB, in addition to coinfection with HIV and in transplant patients.^6^ The disease occurs predominantly in the population over 50 years of age and is related to the presence of additional comorbidities.^6^

An accurate diagnosis that identifies pulmonary disease caused by NTMs is a challenge in clinical practice,^7^ since, in addition to nonspecific symptoms, infections can originate from colonisation and transient contamination.^8^ Thus, proper treatment and resolution of the disease depend on a diagnosis that associates clinical findings with laboratory tests.^4^ ^,^ ^8^ According to guidelines established by the American Thoracic Society (ATS), European Respiratory Society (ERS), European Society of Clinical Microbiology and Infectious Diseases (ESCMID), and the Infectious Diseases Society of America (IDSA), at least two cultures of NTMs are required for evidence of disease, and susceptibility testing is recommended due to the presence of resistance in relevant species,^9^ which makes rapid identification of NTMs important for patient management and disease control.^4^ ^,^ ^8^

The identification of NTMs is carried out mainly by phenotypic methods based on culture and biochemical characteristics, which makes the process time-consuming and laborious.^10^ ^,^ ^11^

The molecular techniques for identifying the species of NTMs that are being used are mainly based on the polymerase chain reaction (PCR), such as *GenoType* ^*®*^ *Mycobacterium CM* and *PRA-hsp65*, which have the advantage of quickly obtaining results, however, the high cost and/or complexity of the process limit its use in the routine.^12^

This study was developed to detect DNA from NTMs by real-time PCR (qPCR) in lung samples, with the aim of differentiating from the *M. tuberculosis* Complex and, additionally, to identify the species through sequencing for comparison of results.

## SUBJECTS AND METHODS

*Clinical isolates (culture) and lung samples of NTMs* - All samples used in the study (68 sputum samples) were from patients at the Phthisiology Outpatient Clinic of Hospital Sanatório Partenon (HSP), a state reference (Rio Grande do Sul, Brazil) for cases of NTMs, treated in the period from September 2018 to December 2019.

Clinical suspicion, as well as diagnosis, followed the criteria established in international guidelines.^9^ The study was approved by the Ethics Committee on Health Research of the Escola de Saúde Pública (CEPS-ESP) in accordance with the Resolution 466/2012, under the number CAAE 96556418.4.0000.5312 of September 24, 2018.

The smear slides, culture and GeneXpert/RIF tests were performed in the diagnostic routine of the HSP, as recommended.^11^ ^,^ ^13^ Patients with an insufficient sample (less than 500 µL) for all tests were excluded from the study, in addition to those being monitored for TB control and responding to treatment. The NTMs strains used as controls in this study came from the Institute of Biological Sciences of Universidade Federal do Rio Grande (Rio Grande, RS, Brazil).

*DNA extraction* - The extraction of DNA directly from culture samples and lung samples was performed by the method known as “sonication”.^14^ The concentration of DNA extracted from the cultures was determined by spectrophotometry using the *Eppendorf BioSpectrometer* ^*®*^ *basic* equipment (Hamburg, Germany).

The study was divided into three stages: standardisation of the qPCR technique with control strains; detection of NTM DNAs in lung samples using the standardised technique and sequencing of the positive samples.

*qPCR standardisation from clinical isolates* - A qPCR (TaqMan^®^ hydrolysis linear probes) methodology was standardised for amplification of the 16S rRNA region^15^ in the 7500 equipment (Applied Biosystems). For this, 11 DNAs from NTMs extracted from culture and confirmed by sequencing were used, as described in item C (Table I).

Primer efficiency was evaluated from a serial dilution using known amounts (100 ng) of *M. avium* DNA. The detection limit was defined using known concentrations of DNA from cultured *M. avium* and serially diluted, in ultrapure water, in factor 10 (16 ng to 0.016 fg) (Figure).

**Figure:**
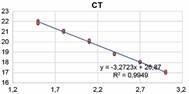
Graph of the analysis of the detection limit and evaluation of the technical efficiency (This figure has not been previously published in any journal).

Assay validation was performed in triplicate with six mixtures of different concentrations, starting with primers and probes at 10 ng (oligonucleotide sequences are based on the study by Kim et al.^15^). Analysing the amplification profile from the curve formed and based on the level of detectable fluorescence (threshold). The smallest amount of primers and probe with the best performance was considered as the standard for the reaction.^16^

*Detection of NTMs by qPCR in a lung sample* - The qPCR for amplification of 16S rRNA from NTMs was performed as standardised, using DNAs extracted directly from sputum samples, by sonication, as described above. The positive control (PC) was a DNA from *M. avium* and the negative control (NC) was ultrapure water. After carrying out the assay using 16S rRNA as a target, the extracted DNAs were amplified by PCR using primers that amplify regions of the IS6110 to identify the presence of *M. tuberculosis* complex DNA or mixed infections.^17^ The reactions used *M. tuberculosis* DNA from the reference strain H37Rv as PC and ultrapure water as NC.^18^

The analysis of the amplification curve followed the predefined parameters (threshold and baseline). An evaluation of the methodology was performed by the amplification of DNAs from previously genotyped mycobacteria.

*Molecular identification of NTMs species by sequencing* - Molecular identification of NTMs in qPCR-positive samples was performed by sequencing the *rpoB* and *hsp65* genes after PCR amplification with primers described by Telenti and coworkers.^19^ PCR for the *rpoB* gene was performed with an initial denaturation step at 94ºC (5 min), proceeding with 40 cycles of denaturation at 94ºC (90 s), hybridisation at 65ºC (2 min), extension 72ºC (3 min) and a final extension cycle at 72ºC (10 min). The PCR for the *hsp65* gene follows the model described above, changing to 45 cycles of denaturation at 94ºC (1 min), hybridisation at 60ºC (1 min) and 72ºC extension (1 min). The reactions were performed on the Applied Biosystems^®^ Veriti^®^ 96-Well ThermalCycler StepOne equipment. As controls, DNAs from NTMs previously identified by culture and confirmed in the sequencing were used (Table I) and as NC, ultrapure water. After checking the DNA amplifications on an agarose gel, the PCR amplicons were subjected to purification by polyethylene glycol (PEG) described by Rosenthal et al.^20^ Cycling with the Big Dye^®^ Kit version 3.1 (Applied Biosystems) was performed according to the manufacturer’s instructions.^21^

**TABLE I:** Comparison of results between culture, real-time polymerase chain reaction (qPCR) and sequencing tests for Non-tuberculous mycobacteria (NTMs)

Sample n**º**	NTM culture	qPCR	Sequencing
1	*M. kansasii*	+	*M. kansasii*
2	*M. fortuitum*	+	*M. fortuitum*
3	*M. szulgai*	+	*M. szulgai*
4	*M. malmoense*	+	*M. malmoense*
5	*M. abscessus*	+	*M. abscessus*
6	*M. marinum*	+	*M. marinum*
7	*M. peregrinum*	+	*M. peregrinum*
8	*M. chelonae*	+	*M. chelonae*
9	*M. gordonae*	+	*M. gordonae*
10	*M. chitae*	+	*M. chitae*
11	*M. avium*	+	*M. avium*

+: positive (16S target detection).

The PCR products were labeled with 5 pmol of the TB11 primer (5’-ACCAACGATGGTGTGTGTCAT-3’, for the *hsp65* gene) or with 5 pmol of the MycoF primer (5’-GGCAAGGTCACCCCGAAGGG0-3’, for the *rpoB* gene), together with 1 µL of reagent (BigDye Terminator v3.1 Cycle Sequencing Kit; Applied Biosystems), to 4.5 µL of purified PCR product in a final volume of 10 µL. Labeling reactions were performed in a 96-well thermal cycler (Veriti; AppliedBiosystems). The sequencing was performed in a genetic analyser from Applied Biosystems^®^ 3130/3130xl Genetic Analysers (Data Collection Software 4).

The sequences obtained were analysed using the Laser gene SeqMan software (DNASTAR, Madison, USA), and aligned through the basic local alignment search tool (BLAST) with other sequences deposited in GenBank (National Centre for Biotechnology Information - NCBI - http://blast.ncbi.nlm.nih.gov).^8^ The sequences were analysed considering the greatest coverage and identity of the species, with a mean coverage of 98.8%.

*Statistical analysis* - Statistical analysis was carried out using the statistical program Statistical Package for Social Sciences - SPSS v.21 (SPSS Ins. Chicago, IL, USA). The agreement between the tests, when performed with clinical isolates, was analysed by the *Kappa* test.^22^

## RESULTS

*qPCR standardisation* - The primers used in the assay for detection of NTMs (16S rRNA) showed an efficiency of 98% (0.988), as shown in Figure. The efficiency was calculated from the reaction slope of -3.35, which is the linear coefficient of the standard curve, indicating the regression coefficient of the curve, showing the efficiency of the reaction amplification. Reproducibility of duplicates (R2) was 0.998.^23^

The qPCR detection limit was 160 pg of DNA of the NTM (*M. avium*) [corresponding to cycle threshold (CT) 30]. The standardisation of the primer concentrations in the validation was 0.4 ng and 0.2 ng for the probe, which showed the best performance.

The test reproducibility was confirmed by the DNA amplification pattern of the 11 NTMs used (Table I).

*Detection of NTM DNA in lung samples* - The 68 lung samples analysed consisted of: 54 from patients with suspected mycobacteriosis (NTM) and 14 from patients with TB (tested for acid-fast bacilli - AFB, *M. tuberculosis* culture and GeneXpert/RIF positive) used as negative controls to assess specificity.

In 28/54 (52%), the culture was positive for *Mycobacterium* (NTMs) that do not belong to the *M. tuberculosis* complex. Of these, 27/28 (96.4%) were positive in the qPCR test for NTMs with CT ranging from 12-17 (Table II). All 26 samples negative for NTMs culture and the 14 samples that were from patients with tuberculosis did not have qPCR amplification for NTM (sensitivity and specificity were 100% and 96%, respectively).

**TABLE II:** Results of positive clinical samples in one of the tests culture, acid-fast bacilli (AFB), and real-time polymerase chain reaction (qPCR) and sequencing

Sample nº	AFB	Culture		Threshold cycles	Sequencing
		NTM	*M. tuberculosis*	CT - PCR 16S	
1	+	+	-	14	*M. fortuitum*
2	+	+	-	16	*M. abscessus*
3	+	+	-	16	*M. abscessus*
4	-	+	-	13	*M. gordonae*
5	+	+	-	14	*M. abscessus*
6	-	+	-	14	*M. abscessus*
7	-	+	-	17	M. gordonae
8	+	+	-	16	*M. gordonae*
9	+	+	-	12	*M. avium*
10	-	+	-	16	*M. fortuitum*
11	-	+	-	18	*M. gordonae*
12	-	+	-	14	*M. fortuitum*
13	+	+	-	15	*M. fortuitum*
14	+	+	-	13	*M. abscessus*
15	+	+	-	14	*M. fortuitum*
16	+	+	-	12	*M. gordonae*
17	-	+	-	13	*M. avium*
18	+	+	-	15	*M. gordonae*
19	+	+	-	14	*M. avium*
20	+	+	-	15	*M. avium*
21	+	+	-	13	*M. gordonae*
22	+	+	-	17	*M. gordonae*
23	+	+	-	17	*M. fortuitum*
24	-	+	-	16	*M. abscessus*
25	-	+	-	13	*M.peregrinum*
26	-	+	-	17	*M. gordonae*
27	-	+	-	16	*M. abscessus*
28	+	+	-	-	-

+: positive; -: negative; CT: cycle threshold; NTM: non-tuberculous mycobacteria; PCR: polymerase chain reaction.

All 27 samples positive for the presence of NTM DNA in the qPCR were sequenced. The identified NTM species were *M. gordonae* in 9/27 (33%), *M. abscessus* in 7/27 (26%), *M. fortuitum* in 6/27 (22%), *M. avium* in 4/27 (15%) and *M. peregrinum* in 1/27 (4%) of the samples.

PCR results were also compared with smear slides tests. Eleven (40.7%) samples that were negative by sputum smear microscopy were positive by qPCR. All were positive in culture for NTM. Only one sample, with positive smear slide, did not show qPCR amplification.

The performance of qPCR, performed from DNA from lung samples, compared to culture, showed an almost perfect agreement (Kappa = 0.93), and with the AFB test the agreement was substantial (*Kappa* = 0.62).

## DISCUSSION

Molecular methods for identifying mycobacterial species are more accurate and faster when compared to conventional methods.^8^

The purpose of this study was to detect NTMs directly from lung samples from patients, using a qPCR with specific primers (16S rRNA) only for NTMs, thus being able to rapidly separate these from the *M. tuberculosis* complex. The 16S rRNA gene contains highly conserved and variable regions, being universally used to identify micobactéria.^18^ ^,^ ^24^ In our study, these primers amplified DNA from several NTMs with an efficiency of 98% (90-100%), with a test detection limit of 160 pg for amplification of 100% of triplicates. Similar analytical sensitivity analyses were used in the study by Peixoto and his coworkers.^25^

The standardised qPCR technique showed an almost perfect agreement with the culture (*Kappa* 0.93), which was confirmed by sequencing. Only one sample was positive for culture, but negative for qPCR and sequencing. In all the others, it was possible to detect DNA from NTMs. This negative sample had culture and positive AFB testing. In the hypothesis that it really is an NTM, it is believed that, possibly, this lack of amplification could be related to PCR inhibitors present in the sample, which were not totally removed in the DNA extraction process,^26^ or loss of DNA during extraction,^27^ since the targets (rpoB and hsp65) used in the sequencing also did not amplify.

Agreement with AFB testing was substantial (*Kappa* = 0.62), that is, 11 AFB negative samples were detected by PCR. Greater detection by qPCR was already expected, due to the need for a high bacterial load (around 10,000 bacilli per mL of sample) for sputum smear positivity,^28^ unlike qPCR, where low DNA concentrations of NTMs could already be amplified.^16^ ^,^ ^29^

When testing the 14 samples from TB positive patients, no amplification was detected, which may allow the use of qPCR to differentiate between TB and NTM. The presence of TB/NTM coinfection has been reported,^30^ ^,^ ^31^ ^,^ ^32^ but it was not detected in this study.

These results suggest that the technique may be promising for detecting NTMs directly from lung samples, ruling out a diagnosis of TB and indicating the possibility of NTM infection/disease in the patient. The target used in this study (16S rRNA), together with the *hsp65* gene, has been described for the identification and differentiation of NTMs from the *M. tuberculosis* complex.^16^ ^,^ ^33^ ^,^ ^34^

Sequencing was used to identify species detected as NTMs. This is described by many authors as the molecular gold standard for the identification of mycobacteria, due to its high discriminatory capacity, allowing the characterisation of bacterial species.^35^ ^,^ ^36^ The *hsp65* and *rpoB* genes have been described for this purpose.^8^ ^,^ ^25^ ^,^ ^34^

The most frequently identified NTM species in this study was *M. gordonae* (33%), being a common mycobacterial species.^37^ *M. gordonae* is considered to be a low virulence strain that is also very associated with laboratory contamination; hence the importance of other factors to make the differential diagnosis of disease caused by NTM, thus reinforcing the need for careful procedures in order to differentiate contamination from infection,^4^ ^,^ ^38^ since *M. gordonae* hardly causes disease.^37^ The results of the study by Shin et al.^39^ suggested that the clinical sensitivity of a test may be strains dependent.

*Mycobacterium abscessus* was the second (26%) most frequently identified bacteria, followed by *M. fortuitum* (22%) and *M. avium* (15%). These potentially pathogenic NTM species are among the most frequent related to lung diseases, both in southern Brazil and in other regions of the country.^4^ ^,^ ^8^ ^,^ ^40^

The limitation found in the present study was the small number of samples analysed. Despite this, the results showed a possibility for the rapid identification of NTM, excluding a diagnosis of TB, especially in patients with symptoms and suspicion of TB, facilitating the beginning of early treatment and contributing to reduce the transmission of this disease.

*In conclusion* - The results suggest that the application of the qPCR technique to detect NTMs using 16S rRNA as a target has the potential to detect NTMs and differentiate them, quickly and efficiently, from *M. tuberculosis*. However, it is still necessary to identify the species to assist in the differential diagnosis between disease and contamination, guiding the choice of the therapeutic scheme.
